# H-Ras oncogene counteracts the growth-inhibitory effect of genistein in T24 bladder carcinoma cells

**DOI:** 10.1038/sj.bjc.6602272

**Published:** 2004-12-21

**Authors:** C Li, R-H Teng, Y-C Tsai, H-S Ke, J-Y Huang, C-C Chen, Y-L Kao, C-C Kuo, W R Bell, B Shieh

**Affiliations:** 1 Department of Microbiology and Immunology, Chung Shan Medical University, No. 110, Sec. 1, Chien Kuo N. Rd., Taichung 402, Taiwan, ROC; 2 Department of Biochemistry, Chung Shan Medical University, No. 110, Sec. 1, Chien Kuo N. Rd., Taichung 402, Taiwan, ROC; 3 Institute of Biomedical Sciences, National Chung Hsing University, No. 250, Kuo Kwang Rd., Taichung 402, Taiwan, ROC; 4 Institute of Molecular Medicine, National Cheng Kung University Medical College, No. 1, Ta Hsueh Rd., Tainan 601, Taiwan, ROC; 5 Department of Urology, Chung Shan Medical University, No. 110, Sec. 1, Chien Kuo N. Rd., Taichung 402, Taiwan, ROC; 6 Internal Medicine, Chung Shan Medical University, No. 110, Sec. 1, Chien Kuo N. Rd., Taichung 402, Taiwan, ROC

**Keywords:** H-Ras, bladder transitional cell carcinoma, genistein, microarray profiling gene expression pattern, drug resistance

## Abstract

Among eight human bladder cancer cell lines we examined, only T24 cells were resistant to the growth inhibition effect of genistein, an isoflavone and potent anticancer drug. Since the T24 cell line was the only cell line known to overexpress oncogenic H-Ras^val 12^, we investigated the role of H-Ras^val 12^ in mediating drug resistance. Herein, we demonstrate that the phenotype of T24 cells could be dramatically reversed and became relatively susceptible to growth inhibition by genistein if the synthesis of H-Ras^val 12^ or its downstream effector c-Fos had been suppressed. The inhibition of Ras-mediated signalling with protein kinase inhibitors, such as PD58059 and U0126 which inhibited MEK and ERK, in T24 cells also rendered the identical phenotypic reversion. However, this reversion was not observed when an inhibitor was used to suppress the protein phosphorylation function of PI3 K or PKC. These results suggest that the signal mediated by H-Ras^val 12^ is predominantly responsible for the resistance of the cells to the anticancer drug genistein.

The oncoprotein Ras, a 21 kDa guanine nucleotide-binding protein, is encoded by a member (Harvey-, Kirsten-, and Neural-ras) of the ras proto-oncogene family (Rayter *et al*, 1989; Maculuso *et al*, 2002). Ras transduces signals in discrete intracellular pathways (Marshall, 1995), with the route via raf-1, MEK, ERK, and finally to induce the expression of the immediate early gene c*-fos* for turning on a cascade of downstream genes as the most prominent pathway in regulating vital cellular processes (Rayter *et al*, 1989; Thorburn and Thorburn, 1994; Maculuso *et al*, 2002). Mutational activation transforms Ras into an oncogenic form, as demonstrated by the substitution of glycine by valine at codon 12 in Harvey (H)-Ras (H-Ras^val 12^), results in the loss of intrinsic GTPase function and therefore the protein is constitutively in the active, GTP-bound state and is continuously sending signals for cell growth (Rayter *et al*, 1989; Maculuso *et al*, 2002). Statistics reveal that 10–25% of all human malignancies in clinics were found to harbour a variety of Ras mutations (Barbacid, 1987; Bos, 1989), making Ras one of the most important targets to suppress tumour cell growth (Rinker-Schaeffer *et al*, 1993; Prendergast *et al*, 1996).

According to the recent surveys of the WHO (www.who.int/cancer/resources/i ncidences/en/), bladder cancer is rated within the five most common cancers in males in North America, Northern and Western Europe, and other developed countries. Transitional cell carcinoma (TCC) is a tumour that occurs mostly in the urinary bladder and has been linked to multiple and accumulated aberrations in oncogenes (e.g. *H-ras* mutations) and cancer-suppressor genes (e.g. p53 inactivation), as well as to the allelic loss of specific chromosomal loci (e.g. chromosomes 9q and 11p) (Habuchi *et al*, 1993; Linnenbach *et al*, 1993; Brandau and Bohle, 2001). Mutational activation of the *H-ras* oncogene was first reported (Reddy *et al*, 1982; Tabin *et al*, 1982) in human T24 TCC cell line that played an essential role in urothelial carcinogenesis. Molecular epidemiological studies conducted within different geographic regions or in different races and tumour stages/grades have revealed that up to 84% of bladder TCC carried activated H-Ras (Fontana *et al*, 1996; Hong *et al*, 1996; Vageli *et al*, 1996; Yu *et al*, 1996; Saito *et al*, 1997; Olderoy *et al*, 1998; Przybojewska *et al*, 2000). Therefore, oncogenic activation of H-Ras is an important tumorigenic factor for bladder tumours.

High soybean food consumption, which provides ingestion of a substantial amount of genistein, has been suggested to contribute to the relatively low rates of many cancers in Asian countries (Lee *et al*, 1991; Messina *et al*, 1994). Genistein, an isoflavone with a structure similar to oestrogen (a phyto-oestrogen), possesses potent inhibitory activities against growth factor-associated tyrosine protein kinases (Akiyama *et al*, 1987) and DNA topoisomerase II (Markovits *et al*, 1989). It is also an antioxidant (Naim *et al*, 1976) with the capability of suppressing angiogenesis and endothelial cell proliferation *in vitro* (Fotsis *et al*, 1993). Genistein is a compound with a variety of potential properties that mainly inhibit tumour growth *in vitro* and *in vivo*. In the mid-1990s, genistein was used for the first time to treat and efficaciously inhibit the growth of human bladder tumour cell lines (Lu *et al*, 1996). In subsequent experiments with bladder cell lines derived from superficial (RT4 cells) and invasive (T24 cells) tumour stages that had been transfected with the different genes to express the diverse expression levels of H-Ras and epidermal growth factor receptor (EGFR), this membrane protein has also been associated with tumorigenesis and is frequently clinically detected in bladder tumours (Dangles *et al*, 1997). Genistein and tyrphostin (an EGFR inhibitor) were both found to preferentially inhibit the motility and growth of bladder carcinoma cell lines that overexpressed EGFR (Theodorescu *et al*, 1998). Cancerous cells that harboured only mutated Ras but not overexpressed EGFR were less susceptible to inhibition by these drugs. The authors (Theodorescu *et al*, 1998) thus concluded that the upregulation of EGFR expression, but not oncogenic H-Ras, played a key role in developing tumour-invasive phenotype in T24 cells. In animal model studies, genistein has been consistently demonstrated to reverse the cancerous phenotype of mouse fibroblast (NIH3T3) cells transformed by v-H-Ras or H-Ras^val 12^ (Okura *et al*, 1988; Kuo *et al*, 1994). Zhou *et al* (1998) reported an in-depth study of the inhibitory effects of genistein on bladder tumorigenesis with both murine and human cell lines and in a mouse animal model (Zhou *et al*, 1998). The result of the study demonstrated that the compound inhibited the growth of two mouse and three human bladder cell lines, in a dose-dependent fashion, via cell cycle arrest at the G_2_/M phase and/or apoptosis. Importantly, mouse tumours derived from the transplantation of murine bladder cancer cells also responded to the inhibitory effects of genistein. A similar report (Su *et al*, 2000) published 2 years later also confirmed that genistein induced cell cycle arrest and inhibited CDC2 kinase activity that suppressed bladder tumour cell growth. These data suggest that the drug has potent anticancer effects both *in vitro* and *in vivo*, and, therefore, genistein has become one of the potential therapeutic compounds to treat patients with urinary bladder neoplasms.

Our research (Chen *et al*, 2001; Shieh and Li, 2004) has focused on gene expression alterations in human bladder tumour cells induced by genistein, in the hope to gain insight into the molecular mechanism(s) of growth inhibition mediated by this drug, and the identification of potential genes that may be further evaluated for possible therapeutic application. Herein, we demonstrate that among eight human bladder tumour cell lines (5637 (Fogh, 1978), BFTC905 (Tzeng *et al*, 1996), HT1197 (Rasheed *et al*, 1977), J82 (O'Toole *et al*, 1978), SCaBER (Cheng *et al*, 1995), T24 (Reddy *et al*, 1982; Tabin *et al*, 1982), TSGH-8301 (Yeh *et al*, 1988), and TCCSUP (Nayak *et al*, 1977) ) tested for the susceptibility of genistein-induced growth inhibition, only the activated *H-ras*-harbouring T24 cells were consistently resistant to this drug treatment. From this investigation, we concluded that the H-Ras^val 12^-induced signal transduction pathway was mainly responsible for the resistant phenotype of T24 bladder TCC cells to the anticancer drug.

## MATERIALS AND METHODS

### Materials, cell cultures, and cell proliferation assay

**Figure 1:**
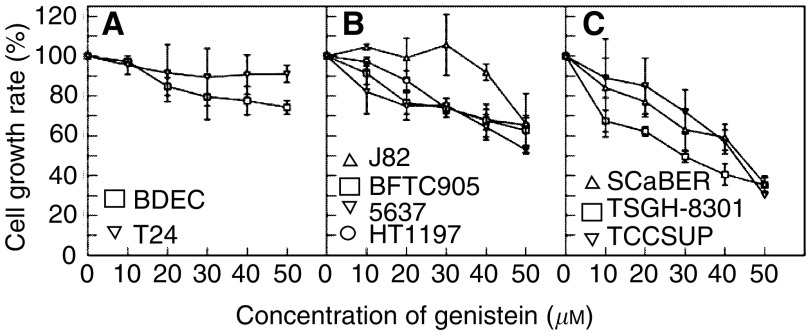
The susceptibilities of bladder TCC cell lines and BDEC to growth inhibition by genistein. The growth inhibition curves for BDEC and eight human bladder TCC cell lines are illustrated. To prevent confusion caused by drawing nine curves with error bars in a small plot, we divided the studied cell lines into three groups (panels **A**–**C**) according to their relative growth rates in the presence of increasing genistein concentrations. Panel **A**: insensitive cell lines include T24 (▿) and primary bladder epithelial cells (□). Panel **B**: 5637 (▿), BFTC905 (□), HT1197 (○), and J82 (▵) were cell lines that were moderately inhibited. Panel **C**: relative sensitive cell lines include SCaBER (▵), TCCSUP (▿), and TSGH-8301 (□). These experiments were performed twice with duplicate samples.

Molecular biology enzymes and reagents were purchased from Stratagene (La Jolla, CA, USA) unless otherwise specified. Standard chemicals and reagents were obtained from Sigma (St Louis, MO, USA). All reagents were used according to the recommendations of the manufacturers. Eight bladder tumour cell lines, including 5637, BFTC905, HT1197, J82, SCaBER, T24, TSGH-8301, and TCCSUP, were all maintained in DMEM supplemented with 10% foetal bovine serum, 100 U ml^−1^ penicillin, and 100 *μ*g ml^−1^ streptomycin (these tissue cultural reagents were from Gibco, Grand Island, NY, USA) under 5% CO_2_ at 37°C. Primary bladder epithelial cells (BDEC) were obtained from BioWhittaker (San Diego, CA, USA) and maintained as recommended by the supplier. Cell proliferation assay with a tetrazolium compound and phenazine ethosulphate (CellTiter 96® AQ_ueous_ One Solution Cell Proliferation Kit, Promega; Madison, WI, USA) was used to analyse the growth-inhibitory effect of genistein (Sigma) according to the protocol reported previously (Chen *et al*, 2001). Each 5000 bladder primary or tumour cells were seeded into wells of 96-well culture plates, followed by addition of genistein in the concentrations indicated in Figure 1. The cells were incubated for 72 h before applying the cell proliferation assays. The genistein stock solution (50 mM) was prepared with pure DMSO (Sigma) and was later diluted to the appropriate concentrations with DMEM at the time of use.

### cDNA microarray and antisense experiments

The experiments of profiling gene expression patterns in different bladder tumour cells treated with 50 *μ*M genistein were routinely performed by using self-produced cDNA microarrays and a hybridisation/detection protocol (Chen *et al*, 2001; Shieh and Li, 2004; w3.csmu.edu.tw/~chingli-Biochi p/). Here, we showed the changes of *egr-1* and c*-fos* expression levels in T24 and TSGH-8301 cells with respect to the time of induction.

Several high-quality phosphorothioate oligodeoxynucleotides (ODNs) were synthesised (MDBio Inc.; Taipei, Taiwan, ROC) to block the synthesis of the target gene products. All these ODNs contained 14 phosphorothioate groups to ensure the best stability during transfection, as demonstrated by the anti-*H-ras* expression experiments reported previously (Monia *et al*, 1996). Except for the *VDUP-1* gene (Han *et al*, 2003), we used the previously reported, as listed below, antisense and control ODNs in experiments targeted to the expressions of *H-ras*, c*-fos*, or *egr-1* gene. They include anti-oncogenic-*H-ras* antisense and controls (antisense, 5′-vCsCsAsCsAsCsCsGACsGsGsCsGsCsCsC-3′; control, 5′-GsAsGsCsTsCsCsCAGsGsCsTsCsAsGsA-3′) (Chen *et al*, 1996; Monia *et al*, 1996), anti-c*-fos* antisense and mutated (antisense, 5′-GsAsAsGsCsCsCsGAGAsAsCsAsTsCsAsT-3′; mutated, 5′-GsAsAsGsTsCsCsAAGGsAsCsGsTsCsAsT-3′, with the underlined nucleotides representing as substitutions) (Gibellini *et al*, 1997), anti-*egr-1* antisense and scrambled (antisense, 5′-vTsGsCsGsGsGsGsCGCGGGsGsAsAsCsAsCsT-3′; scrambled, 5′-GsTsCsGsCsAsCsGCGTGCsGsAsGsGsAsGsG-3′) (Sells *et al*, 1995), and anti-*VDUP-1* gene antisense and sense (antisense, 5′-TsTsCsTsTGAACATCAsCsCsAsT-3′; sense, 5′-AsTsGsGsTGATGTTCAsAsGsAsA-3′) ODNs, where an ‘s’ indicates a phosphorothioate-capped moiety and they were used in cell proliferation assays or immunoblotting experiments. Approximately 6000 appropriate cells were seeded in the wells of a 96-well flat-bottom microtitre plate, followed by adding indicated amounts of an ODN premixed with ESCORT transfection reagent (used according to the instruction provided by Sigma) and incubating in a CO_2_ incubator for 8 h at 37°C. The cells were further treated with 50 *μ*M genistein for another 72 h before they were subjected to cell proliferation assays. As the controls for T24 cell proliferation assays under various conditions, BDEC and TCCSUP cells were also grown and passed through the identical procedure in parallel.

### Reverse transcription–polymerase chain reaction and immunoblotting analyses

The expression levels of *egr-1* and c*-fos* in TSGH-8301 and T24 cells treated without or with 50 *μ*M genistein for the various time points were determined by semiquantitative reverse transcription–polymerase chain reaction (RT–PCR). The amplification primers used were: (1) *β*-actin: forward 5′-ATCATGTTTGAGACCTTCAA-3′ and reverse 5′-CATCTCTTGCTCGAAGTCCA-3′; (2) *egr-1*: forward 5′-CTGCACGCTTCTCAGTGTTC-3′ and reverse 5′-AGCAGCATCATCTCCTCCAG-3′; and (3) c*-fos*: forward 5′-AAGGAGAATCCGAAGGGAAA-3′ and reverse 3′-GCTGCTGATGCTCTTGACAG-3′. The RT–PCR of these genes was performed at the cycles that the amplification of DNA molecules was in the exponential increasing stage. After many tests to correlate with the PCR product amounts and thermocycling numbers (data not shown), we used 20 amplification cycles for *β*-actin and 27 cycles for both *egr-1* and c*-fos* genes. The RT–PCR products were visualised by agarose gel electrophoresis in the presence of ethidium bromide.

When performing Western blot analyses with ODN-treated cells, 5 × 10^6^ cells were placed in a six-well culture plate, followed by treating with ODNs, 5 *μ*M each time, and the same amount of genistein, as in the protocol described above. After incubation and removal of culture supernatants, 50 *μ*l of RIPA lysis buffer (Helfrich *et al*, 1994) was used to lyse and then collect cell extracts for SDS–PAGE. The entire immunoblotting procedure employed a commercial standardised technique (BM Chemiluminescence Western Blotting Kit, Roche Applied Science; Mannheim, Germany) with rabbit antibodies against human H-Ras (clone C-20), c-Fos (4), and Egr-1 (588) proteins that were purchased from Santa Cruz Biotechnology, Inc. (Santa Cruz, CA, USA). Chemiluminescent detections of antibody–antigen complexes revealed the target proteins on X-ray film. The cell proliferation assays and Western blotting experiments were all performed in triplicate and identical results were obtained.

## RESULTS

### The effect of genistein on bladder tumour cell growth and gene expression

In order to extend our previous efforts to gain insights into the molecular mechanism for genistein-induced cell growth inhibition in bladder tumour cells (Chen *et al*, 2001; Shieh and Li, 2004), we tested the effect of the drug on eight bladder tumour cell lines (including 5637, BFTC905, HT1197, J82, SCaBER, T24, TSGH-8301, and TCCSUP) and BDEC. We divided the cell lines into three groups depending on their susceptibilities to the growth-inhibitory effect of genistein: T24 cell line and BDEC were relatively insensitive to the drug treatments that consistently maintained the growth rate above 75% of the respective mock-treated cells when the highest concentration (50 *μ*M) of the drug was used (Figure 1A); cell lines HT1197, HT1376, J82, BFTC905, and 5637 moderately reduced their growth rates to 65–55% (Figure 1B); and SCaBER, TSGH-8301, and TCCSUP cells were always susceptible to the drug treatments as their growth rates were dramatically reduced to 30–35% at the genistein concentration of 50 *μ*M. We also found that the J82 cell line had elevated growth rates at low genistein concentrations (Figure 1B), which might be due to the stimulatory effect of DMSO solvent (10%), a similar effect had been observed with HepG2 and Hep3B hepatocellular carcinoma cell lines (Li, unpublished data), and therefore the growth-inhibitory effect could be detected only when the highest concentration of genistein (50 *μ*M) was used. Since BDEC was not a tumour cell line but was regarded as the normal cell control, the growth should not be greatly inhibited by the anticancer drug, and indeed, the growth rates were only slightly reduced and then maintained close to 80% at concentrations equal to or greater than 20 *μ*M (Figure 1A). The cell proliferation assays demonstrated that T24 was the only TCC cell line that consistently resisted genistein, and the growth rates were always maintained at 90–95% during the drug treatments. Although the degree of the inhibition varied among cell lines, this result basically agreed with the previous results reported by Zhou *et al* (1998).

**Figure 2:**
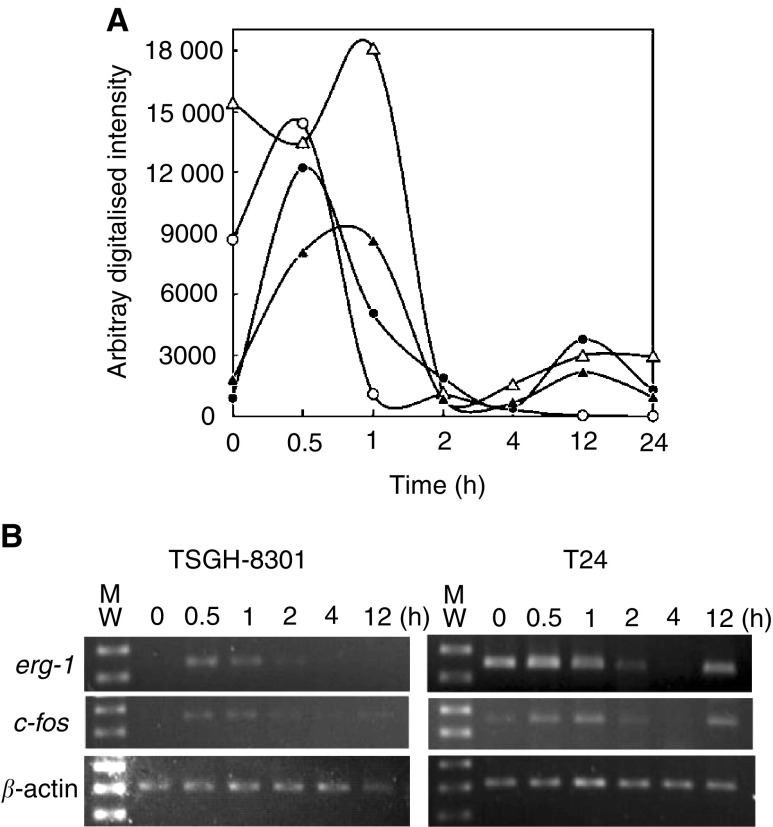
Different expression patterns of *egr-1* and c*-fos* in T24 and TSGH-8301 cells. (**A**) Treatment of T24 and TSGH-8301 cell lines with 50 *μ*M genistein for the indicated periods of time revealed different kinetic patterns for *egr-1* and c*-fos* expressions (the scale on *x*-axis is not in proportion with time). The expression levels for genes, as presented by arbitrary digitalised intensity, were derived from experiments with cDNA microarrays and the subsequent quantification of hybridisation signals according to the protocol described previously (Chen *et al*, 2001; Shieh and Li, 2004). In drug-sensitive TSGH-8301 cells, the basal expressions of *egr-1* (▴) and c*-fos* (•) were minimal, but were induced acutely and transiently after stimulation with genistein. On the contrary, in T24 cells, the genes for Egr-1 (▵) and c-Fos (○) were highly expressed without stimulation, but could be further induced after treatment with genistein, followed by acute decreases to low levels. These experiments were performed in triplicate. (**B**) The expression patterns of both genes have also been confirmed by using RT–PCR with RNA samples from TSGH-8301 and T24 cells stimulated by genistein for 0–12 h. Similar to the microarray experiments, TSGU-8301 cells did not express either *egr-1* or c*-fos* without genistein treatment, whereas T24 cells constitutively expressed these two genes. At 0.5 h post induction with the drug, the expression levels of both genes were upregulated to the highest levels, followed by linear reduction to the levels close to the original production ratio at 12 h. The size of the *egr-1*, c*-fos*, and *β*-actin PCR DNA fragments are 260, 373, and 318 bp, respectively. These experiments were performed in duplicate.

In the previous experiments, we used a self-made Millennia-Chip, version 1 cDNA microarrays to profile differential gene expression patterns in genistein-stimulated TCCSUP, TSGH-8301, and T24 bladder TCC cell lines (Chen *et al*, 2001; Shieh and Li, 2004). The most notable finding in these studies was the dramatic difference in the expression patterns of two immediate early genes *egr-1* and c*-fos*. In the genistein-sensitive cell lines, TCCSUP and TSGH-8301, the genes revealed transient and inducible kinetics which peaked at 0.5 h post treatment of genistein just as were reported in many physiological conditions (Liu *et al*, 1991), whereas in genistein-resistant T24 cells both genes were constitutively expressed but could be induced to the higher levels by the drug, followed by declining to the lower levels. Figure 2A demonstrates the expression levels of *egr-1* and c*-fos* in TSGH-8301 and T24 cells derived from cDNA microarray analyses, whereas Figure 2B confirms such gene expression profiles with RT–PCR. Since the overexpression of oncogenic H-Ras^val 12^ has only been reported in T24 cells, we speculated that the oncoprotein stimulated the coincidental abnormal *egr-1* and c*-fos* expression patterns in the cells, as reported previously (Stacey *et al*, 1987; Alexandropoulos *et al*, 1992). Therefore, we investigated the role of H-Ras^val 12^ in mediating cellular signal transduction pathway in T24 tumour cell line that rendered the resistant phenotype against the growth-inhibition effect of genistein.

### *In vitro* targeting gene expressions and the susceptibility to genistein inhibition

**Figure 3:**
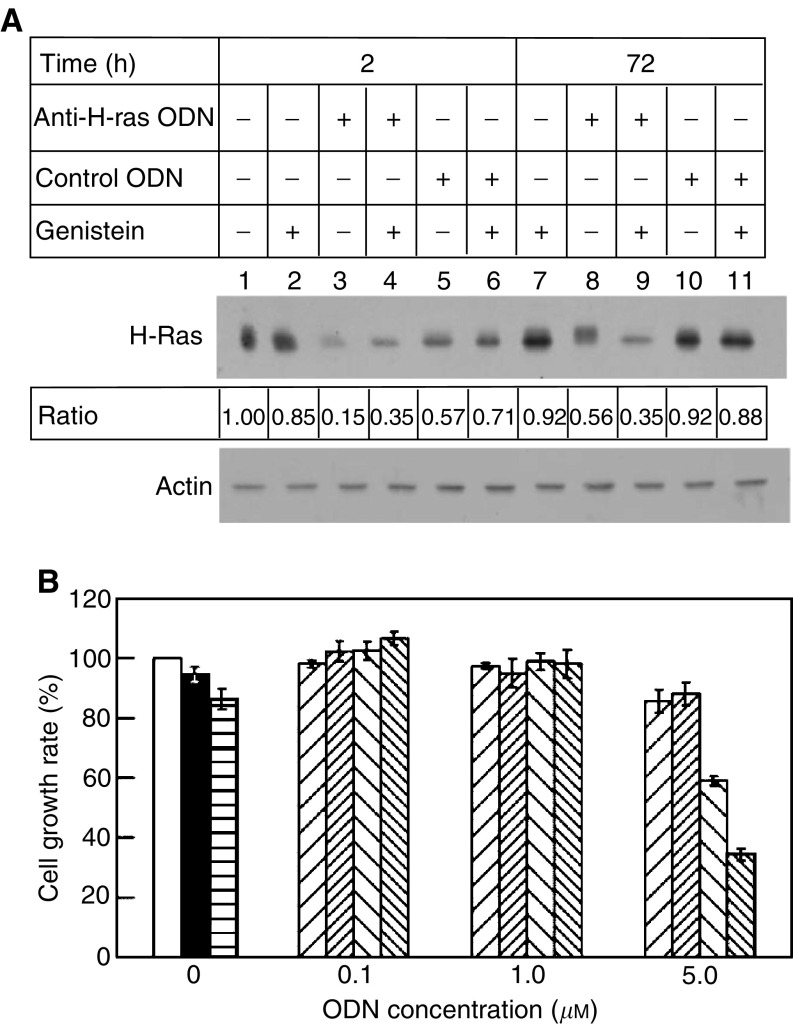
Influence of H-Ras^val 12^ ovexpression on the growth of T24 cells responding to antisense oligonucleotide and genistein treatments. (**A**) T24 cells were treated with 5 *μ*M of the indicated phosphorothioate ODN alone or the ODN and 50 *μ*M genistein, followed by cell extract isolation and Western blotting with the anti-H-Ras antibody. The intensities of H-Ras^val 12^ protein were quantified with a densitometer, and the Ratio line revealed the percentage of the protein produced (after normalising with the levels of actin protein) in the ODN or/and genistein-treated cells *vs* untreated T24 cells. The result revealed that the expression of H-Ras^val 12^ was inhibited to a greater degree by treating with the antisense ODN than with the control ODN or genistein, and the effect was strikingly obvious after 72 h incubation. Under all experimental conditions, actin remained relatively unchanged. The control ODN was a 17-mer targeted to human immunodeficiency virus, which was used in parallel with anti-*H-ras*^val 12^ ODN, as described previously (Chen *et al*, 1996). (**B**) With increasing amounts of different ODNs, the growth rates of T24 cell line were determined in the presence or the absence of 50 *μ*M genistein. The plot reveals that only when the anti-*H-ras*, but not control, ODN (5 *μ*M) is added, the growth of T24 cells is inhibited in the absence of genistein and the growth rate is further reduced to below 40% of the untreated cells when the drug is added, suggesting that the expression of H-Ras renders drug-resistant phenotype. The symbols used are: cells without any treatment, □; and cells treated with 0.5% DMSO solvent, ▪; 50 *μ*M genistein, 
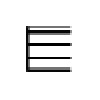
; the control ODN, ▨; the control ODN supplemented with 50 *μ*M genistein, 
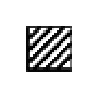
; the anti-H-ras antisense ODN, ▧; and the antisense ODN plus genistein, 
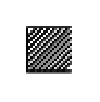
.

Since the most prominent signal transduction pathway regulated by Ras is Raf-1-mediated signalling which results in c-Fos induction (Maruta and Burgess, 1994), we examined the effect of H-Ras^val 12^ or c-Fos expression on the susceptibility of T24 cells to genistein inhibition. Antisense phosphorothioate ODNs targeted to the expression of either gene was used to treat T24 cells, followed by assaying the cell proliferation rate in the presence of 50 *μ*M genistein. The experiments detected that the levels of H-Ras^val 12^ were moderately reduced in the cells treated with 5 *μ*M of control ODN for 2 h (Figure 3A, lanes 5 and 6 *vs* lanes 1 and 2, respectively), whereas the levels fell dramatically to one-third or lower of the original level if the same amount of the anti-*H-ras* ODN was used for the same period of time (Figure 3A, lanes 3 and 4 *vs* lanes 1 and 2, respectively), as determined by immunoblotting. After 72 h incubation, the effect of anti-H-ras ODN on blocking H-Ras^val 12^ synthesis was even more obvious (lanes 8 and 9 *vs* lanes 10 and 11, respectively). In all studies, the presence or absence of genistein did not affect the expression of H-Ras greatly or yielded inconsistent expression levels (odd *vs* even samples in lanes 1–6 and also in lanes 8–11). Since the anti-*H-ras* ODN could effectively block the gene expression level, H-Ras^val 12^ downregulated T24 cells that were subsequently subjected to the cell proliferation assay. Figure 3B reveals that when the various amounts of the control ODN were used, the growth rates of T24 cell line only decreased slightly regardless of whether genistein is present or absent, whereas the cell growth rate is moderately reduced (to 60% of untreated cells) when the cells are no longer overexpressing oncogenic H-Ras^val 12^ (at 5 *μ*M anti-*H-ras* ODN concentration). Upon treatment of the cells with both anti-*H-ras* ODN and 50 *μ*M genistein in cell culture, the T24 cell growth rate was further decreased to the level (about 35%) that was comparable to the rates of the genistein-sensitive bladder TCC cell lines, SCaBER, TSGH-8301, and TCCSUP (Figure 1C). The specificity of the growth inhibition by antisense ODN was further fortified by using a mutated anti-*H-ras* ODN (5′-CsCsGsCsAsCsCsGTCsGsGsAsGsCsCsC-3′, with the underlined nucleotides representing substitutions) (Monia *et al*, 1996) to treat T24 cells. In this experiment, the growth rates were similar to those treated with control ODN in the presence or absence of genistein (data not shown). The result of this antisense experiment indicates that, without H-Ras^val 12^ overexpression, T24 cells become susceptible to the growth inhibition mediated by genistein.

**Figure 4:**
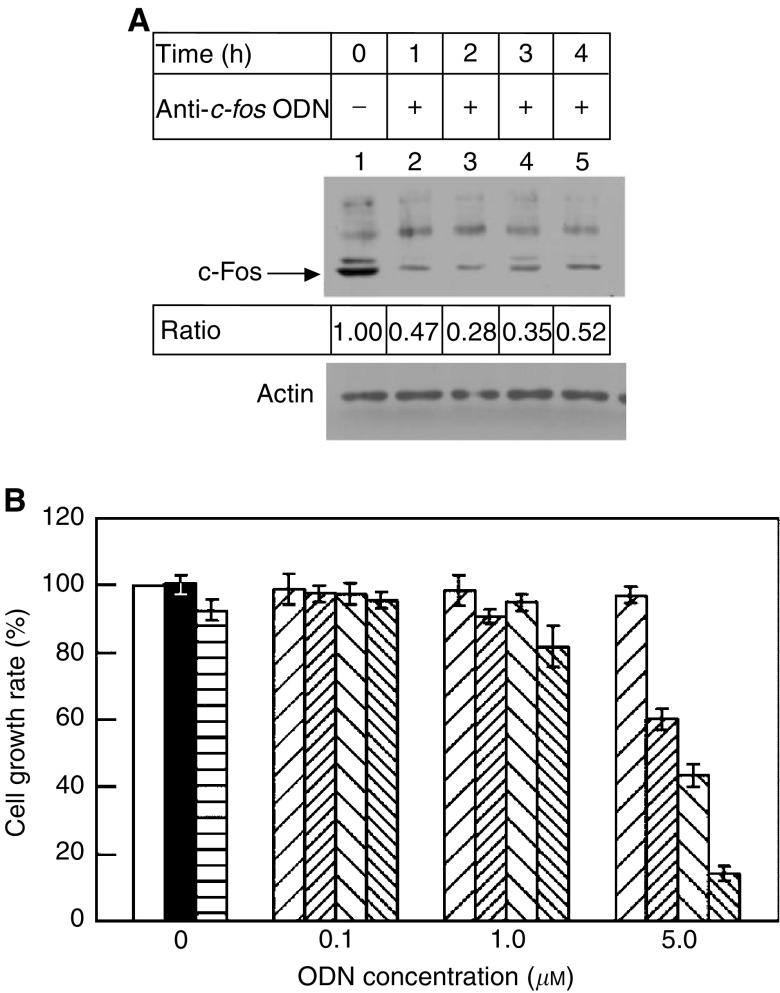
Influence of c*-fos* expression on the growth of T24 cells responding to antisense ODN and genistein treatments. (**A**) The level of c-Fos was highly expressed in mock-treated cells (lane 1), whereas the c-Fos protein level was greatly reduced 1 h after the anti-c*-fos* ODN (5 *μ*M) was added and the suppression lasted for several hours (lanes 2–5). The ratio line indicates the percentage of the protein produced in the ODN or/and genistein-treated cells *vs* untreated T24 cells. Under all experimental conditions, the levels of actin protein remained unchanged. (**B**) This plot demonstrates that the growth of T24 cells is not inhibited when adding the mutated ODN up to the concentration of 5 *μ*M, but the growth rates are greatly decreased, to essentially 40% of the mock-treated cells, when the same amount of the anti-c*-fos* ODN is present in cell cultures. Adding 50 *μ*M genistein to T24 cells pre-treated with 5 *μ*M mutated ODN affected the growth rate moderately (to about 60%), but the cells almost ceased to grow (to 10–15%) as the antisense ODN treatments (5 *μ*M) were combined with genistein. As an increased amount of the ODNs (10 *μ*M) was applied to the identical experiments described above, a similar result was obtained (data not shown). The symbols used here are: cells without any treatment, □; and cells treated with 0.5% DMSO, ▪; 50 *μ*M genistein, 
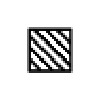
; the mutated ODN, ▨; the mutated ODN supplemented with 50 *μ*M genistein, 
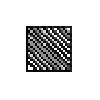
; the anti-c*-fos* antisense ODN, ▧; and the antisense ODN plus genistein, 
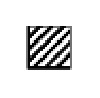
.

It is well known that c-Fos is the downstream effector gene of Ras, and any signal transducing through the Ras/Raf-1 signalling pathway results in nuclear induction of c-Fos, a subunit of the transcription factor AP-1. We intended to test the drug sensitivity of T24 cells when the *c-fos* expression had been retarded *in vivo* by treating with the ODN that has been reported to be effective in blocking cellular c-Fos expression (Gibellini *et al*, 1997). We detected that when 5 *μ*M anti-c*-fos* ODN was added to T24 cells, the levels of c*-fos* were dramatically decreased to half of the detected level in untreated cells at all time points (Figure 4A, lane 1 *vs* other lanes), and the drug-resistant phenotype of T24 cells was reversed (Figure 4B). In these experiments, we used a mutant anti-c*-fos* ODN, as a negative control, because it contained a sequence like the anit-c*-fos* ODN, but with four dispersed nucleotide substitutions that could interrupt the specific binding to its target c*-fos* and lost inhibitory capability. The use of the mutated ODN can thus demonstrate the specificity of antisense oligonucleotide in blocking the expression of c-Fos. As detected in Figure 4, the mutant anti-c*-fos* ODN and genistein together inhibited cell growth moderately (to about 60%), whereas 5 *μ*M anti-c*-fos* ODN alone was able to reduce T24 cell growth rate to 40% of the mock-treated cells, which might be due to the efficient inhibition of c-Fos expression and activity that led to reduction in the growth rate. The growth rate of the T24 cell line was further decreased to 15% when both anti-c*-fos* ODN and genistein were employed simultaneously. Similar results were also observed when 10 *μ*M of all ODNs was used to treat cells under identical conditions (data not shown), suggesting that the correlation between the treatment with antisense or mutated ODN and the drug-resistant phenotype of T24 cells is specific. We thus concluded that c-Fos expression also influenced the drug susceptibility of T24 cells, just as its upstream regulator H-Ras^val 12^. Although Ras and c-Fos are in the same pathway, the inhibitory effect of c*-fos* ODN was greater than that of ras ODN, which may be represented by the differential efficiency of individual ODNs on inhibiting their targets, and that frequently depends on the nature and/or nucleotide sequences of the molecules.

**Figure 5:**
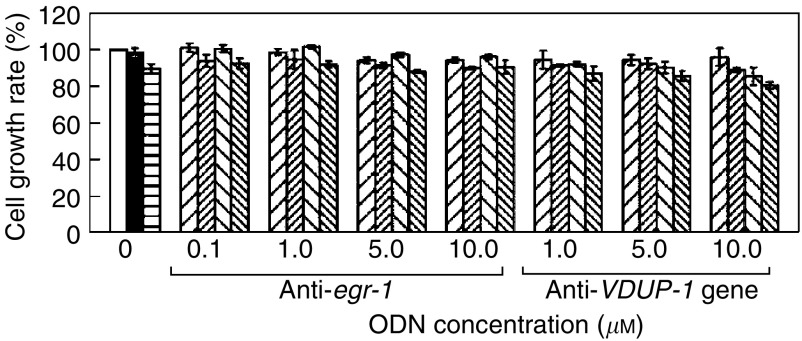
Suppression of *egr-1* or the *VDUP-1* gene expression did not affect the growth of T24 cells treated with genistein. Since the expression of the *egr-1* and *VDUP-1* genes was dramatically induced by genistein in T24 cells, their role in reversing the inhibitory effect of the drug was investigated. The effect of blocking gene expression by the antisense and their control ODNs was tested with Western blotting, and expected results were obtained (data not shown). The result of cell proliferation assays revealed that, regardless of the presence and absence of 50 *μ*M genistein, treatment of the T24 cell line with the antisense ODN for either gene (up to 10 *μ*M), did not alter the cell growth pattern, suggesting that these proteins are not involved in the drug resistance mechanism. In these experiments, the nucleotide sequence-scrambled *egr-1* ODN, which had been characterised previously (Sells *et al*, 1995) and the sense-strand ODN for the *VDUP-1* genes, which is according to the reported gene sequence (Han *et al*, 2003), were used as controls. The result demonstrated that they did not have any influence on the growth of T24 cells even when genistein was added. The symbols used here are: cells without any treatment, □; and cells treated with 0.5% DMSO, ▪; 50 *μ*M genistein, 
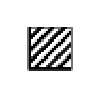
; the control ODN for *egr-1* or the *VDUP-1* gene, ▨; the control ODN for *egr-1* or the *VDUP-1* gene supplemented with 50 *μ*M genistein, 
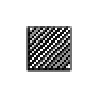
; the antisense ODN for either one gene, ▧; and the antisense ODN for either one gene plus genistein, 
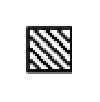
.

The specificity of the above antisense experiments was further investigated. From the previous microarray hybridisation experiments, the *egr-1* and *VDUP-1* gene expressions were known to be significantly induced by genistein in TCCSUP and TSGH-8301 cells (Chen *et al*, 2001; Shieh and Li, 2004), and the former gene was constitutively overexpressed in T24 cells (Figure 2). Since alterations in gene expression patterns frequently indicate the involvement of the gene products in the biological/pathological processes of interest, we examined the role of *egr-1* or *VDUP-1* in the resistance of T24 cells to genistein, and the results were compared to those from the antisense ODN experiments (Figures 3 and 4) studying the functions of H-Ras^val 12^ and c-Fos. Our experiments detected that, regardless of the *egr-1* antisense or the scrambled control ODN used in cell cultures, the drug sensitivities of T24 cells remained unchanged, and the same results were also obtained from experiments using antisense or sense ODN targeted to the expression of the *VDUP-1* gene (Figure 5). These data suggest that the influence of H-Ras or c-Fos downmodulation on the drug susceptibility of T24 cells was specific. We therefore concluded that the resistance of T24 cells to the genistein growth-inhibitory effect must involve the H-Ras^val 12^/c-Fos-mediated signal transduction pathway.

### Influence of the functions of proteins kinases on the growth of T24 cells

**Figure 6:**
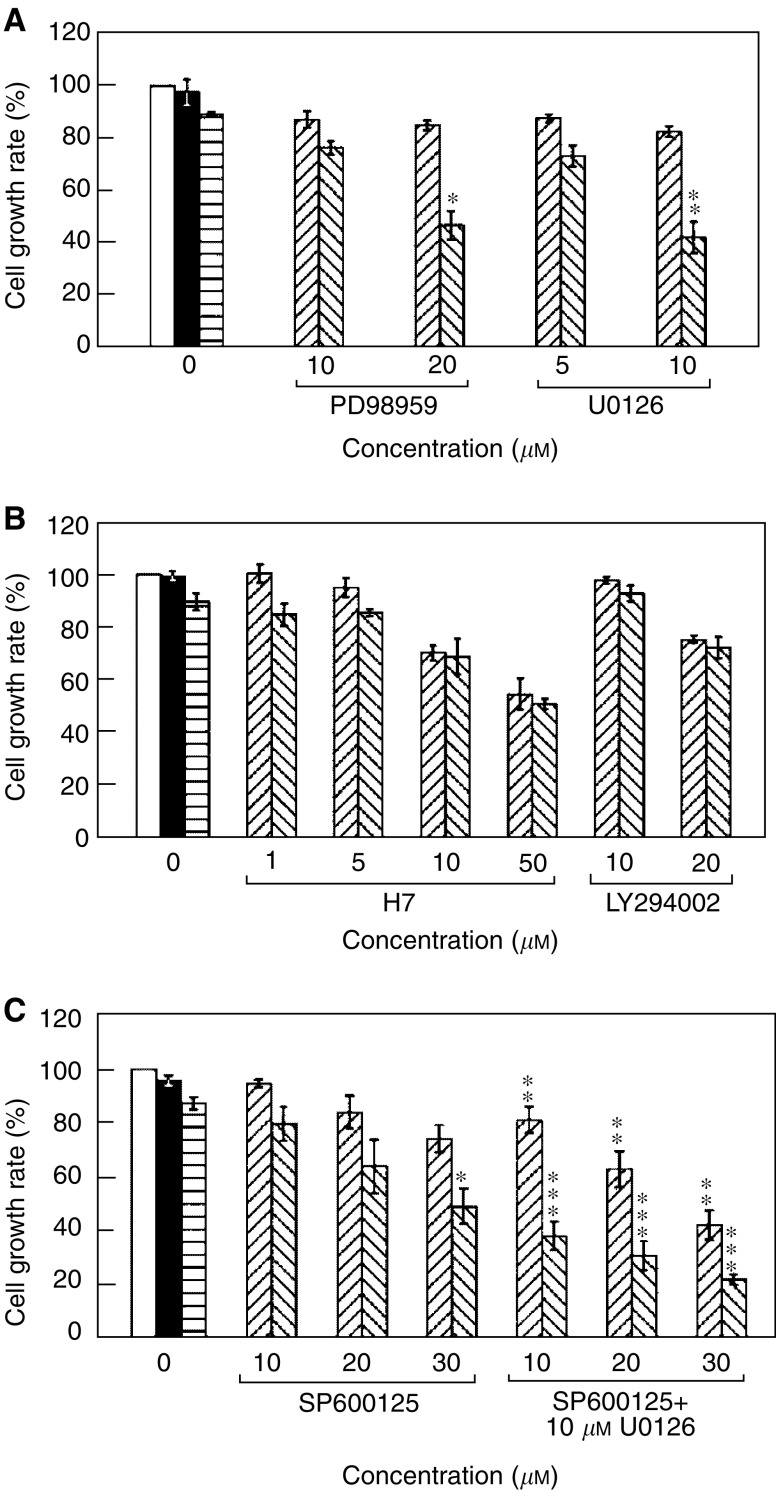
Suppression of T24 cell growth with protein kinase inhibitors and genistein. (**A**) When 50 *μ*M genistein and either PD98059 or U0126 were applied to T24 cells simultaneously, the cell growth rates were reduced in a dose-dependent manner. The difference reached a significance level at 20 *μ*M PD98059 (marked by ^*^) or 10 *μ*M U0126 (marked by ^**^), as compared to that of the cells treated only with the respective inhibitor without genistein (both *P*<0.05 by *t*-test). (**B**) Other protein kinase inhibitors such as H7 (PKC inhibitor) and LY294002 (PI3 K inhibitor) did not reverse the T24 drug resistance phenotype but exhibited only cytotoxicity, as H7 or LY294002 alone could also reduce cell growth rates to the levels similar to the cell cultures simultaneously treated with the inhibitors and the drug at the high concentrations. (**C**) Adding JNK inhibitor SP600125 to T24 cells rendered the dose-dependent growth suppression in the presence of genistein. Statistical analysis (*t*-test) revealed that the reduction of the cell growth rate reached significance (*P*<0.05) only when genistein was added to T24 cells that were previously treated with 30 *μ*M SP600125 (*) as compared to that of the cells treated only with SP600125. The dose-dependent suppression of cell growth was even more substantial when inhibitors for both JNK, at the concentrations of 10, 20, or 30 *μ*M, and MEK/ERK (U0126) were simultaneously added to T24 cell cultures, as compared to those cells treated only with 10, 20, or 30 *μ*M SP600125, respectively (all *P*<0.05 and indicated by ^**^). Furthermore, comparing the growth rates of T24 cells pretreated only with 10 *μ*M U0126 and the indicated amounts of SP600125, the rates were again significantly reduced when the anticancer drug was simultaneously applied (all *P*<0.05 and indicated by ^***^). In this experiment, the growth rate reached levels comparable to those of the genistein-sensitive cell lines. The symbols used in all panels are: cells without any treatment, □; and cells treated with 0.5% DMSO, ▪; 50 *μ*M genistein only, 
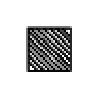
; cells only treated with protein kinase inhibitor(s), ▨; cells treated with both protein kinase inhibitor(s) and genistein, ▧.

The ras gene product regulates a number of discrete intracellular pathways through its different downstream effectors such as Raf-1, RalGDS, and PI3 K (Maruta and Burgess, 1994; Ramirez *et al*, 2002), and probably crosstalks to the JNK activation pathway (de Ruiter *et al*, 2000). To confirm the H-Ras activation in T24 cells rendered resistant to the anticancer drug genistein, protein kinase inhibitors were used to dissect Ras-regulated signal transduction pathways. First, PD58059 and U0126 were employed to inhibit the signal transduced from H-Ras to c-Fos by blocking the kinase functions of MEK and ERK. Figure 6A shows that T24 cells became genistein-sensitive as the growth rates were significantly decreased when 50 *μ*M genistein and either PD58059 (20 *μ*M) or U0126 (10 *μ*M) are present (both *P*<0.05 by *t*-test, two-tailed), and both inhibitory effects were in a dose-dependent manner. This result was completely in agreement with the previous (Figures 3 and 4) antisense experiments with the anti-*H-ras* and anti-c*-fos* ODNs. Protein kinase inhibitors H7 and LY294002, which block the signalling pathways associated to PKC and PI3 K, respectively, were also used in T24 cell proliferation assays with and without the presence of 50 *μ*M genistein. We detected that, at H7 concentration equal to 10 *μ*M or greater, the growth rates of T24 cells were decreased regardless of the presence or absence of the drug, suggesting that H7 did not influence the drug susceptibility, and the cytotoxicity was responsible for cell quiescence at the higher H7 concentrations (Figure 6B). The inhibitor LY294002 at the effective concentration of 20 *μ*M could only slightly reduce the T24 cell growth rate from 95 to 75% of the mock-treated cells in the presence and absence of genistein. The results rule out the involvement of the PKC or PI3 K signal transduction pathway in the drug resistance of the T24 cells.

The Ras pathway is known to interact with the JNK pathway, as the Ras downstream effector c-Fos forms the transcription factor AP-1 with c-Jun, a nuclear protein regulated by the function of JNK (de Ruiter *et al*, 2000). Owing to this, we investigated the role of JNK in the susceptibility of T24 cells to genistein. Figure 6C demonstrates that the JNK inhibitor SP600125 itself exhibits a mild dose-dependent inhibitory effect on T24 cell growth, and the addition of 50 *μ*M genistein potentiates the growth suppression further down to the significant lower level, about 50% of the mock-treated cells, at a concentration of 30 *μ*M SP600125 used (*P*<0.05 by *t*-test, two-tailed), and the level is similar to that by 20 *μ*M PD98059 or 10 *μ*M U0126 as demonstrated in Figure 6A. Since the JNK inhibitor alone only partly inhibited cell growth, dual protein kinase inhibitors were tested. The dose-dependent growth inhibition was again observed if both inhibitors of the H-Ras (U0126) and JNK (SP600125) signalling pathways were used together. As illustrated in Figure 6C, treatment of T24 cells with 10 *μ*M U1026 and increasing amounts of SP600125 (10–30 *μ*M) renders the decreasing T24 cell growth rates down to 50% of untreated cells (all reached significant levels at *P*<0.05). Under identical culture condition, addition of genistein further inhibited the growth rates significantly to approximately 20% of the original growth rate (all *P*<0.05), which was comparable to those of the genistein-sensitive bladder TCC cell lines (Figure 1). These data suggest that signal transduction through the JNK pathway is also involved in the resistance of T24 cells to genistein.

## DISCUSSION

In this study, we employed microarray technology, antisense oligonucleotide inhibition, and cell proliferation assays to identify and characterise genes that are playing key roles in the resistance of T24 bladder TCC cell lines to the anticancer drug genistein. Genistein is a phyto-oestrogen that is present in high quantity in many traditional Asian diets containing soybean products (Messina *et al*, 1994). The compound is regarded as a potential and ideal anticancer drug to treat patients with bladder tumours, because the compound is a natural product present in diet that has been proven to be effective in treatment of bladder TCC (Zhou *et al*, 1998; Su *et al*, 2000), as well as safe with minimal side effects and inexpensive. In 1996, the compound was subjected to the clinical chemoprevention trials for preventing breast cancer and acute lymphoblastic leukaemia (Quella *et al*, 2000; Wang, 2000). Although genistein has shown some promising cancer cell growth-inhibitory effects, many concerns, such as which function(s) is (are) actually playing an essential role in tumour suppression or does it involve the induction of gene expression, remain to be determined. We therefore rationalised that a functional genomic study of genistein-induced gene expression alternation would be the most effective and also the most rapid means to identify the key gene(s) involving the cell growth regulatory pathway.

The anticancer effect of genistein has been mostly studied in breast tumours at the molecular level (El-Deiry *et al*, 1993; Harper *et al*, 1993), and the regulatory event was independent of the oestrogen receptor and the cancer suppressor p53 protein (Li *et al*, 1999; Xu and Loo, 2001). Bladder TCC has been shown to undergo apoptosis induced by genistein through the EGFR signalling pathway involving p21^WAF1/CIP1^ expression (Dangles *et al*, 1997). It has also been demonstrated that genistein exhibited dose-dependent (0–50 *μ*M) growth inhibition of many murine and human bladder cancer cell lines, providing evidence of cell growth arrest at the G2/M phase followed by apoptosis in some cell lines (Zhou *et al*, 1998). In this study, we focused on the genistein growth inhibition of only human cell lines, and compared different gene expression patterns in human cell lines sensitive and resistant to the genistein treatments with cDNA microarrays. From the results, we are convinced that the overexpression of oncogenic H-Ras^val 12^ and its control of the signal transduction pathway were solely responsible for the resistance of the T24 bladder TCC cell line to genistein growth inhibition. It is possible that genistein inhibits the growth signals from the tyrosine protein kinase-associated receptors, such as EGFR and PDGFR (platelet-derived growth factor receptor), to small G protein Ras and thus suppresses cell growth, just like the inhibitory model proposed for genistein prevention of phenylephrine-induced Ras activation (Thorburn and Thorburn, 1994). In such a situation, T24 H-Ras^val 12^, which is downstream from the action site of genistein, constantly sends very strong cellular proliferative signals, resulting in the drug-resistant phenotype. H-Ras^val 12^ is also responsible for maintaining the fast growth rate of T24 cells in routine tissue cultural experiments, and blocking the protein or c-Fos synthesis with antisense OND or interrupting the function of Ras downstream protein kinases by inhibitors frequently resulted in decreasing the growth rate, as we observed in many of our cell proliferation experiments (Figures 3B, 4B and 6C). However, it is still unclear why other TCC cell lines such as TCCSUP and TSGH-8301 could be induced to transiently express c-Fos but remained susceptible to the inhibitory effect of genistein. Furthermore, our finding that the overexpression of the *H-ras* oncogene counteracts the anticancer effect of genistein in T24 cells is contrary to previous reports demonstrating that the drug was capable of (1) reversing the malignant phenotypes of mouse fibroblast (NIH3T3) cells transformed by v-H-Ras or H-Ras^val 12^ (Okura *et al*, 1988; Kuo *et al*, 1994; Li, unpublished data) and (2) suppressing the Ras activity induced by phenylephrine in neonatal rat ventricular myocytes (Thorburn and Thorburn, 1994). This discrepancy may be due to differences in genetic and tumorigenic backgrounds, as suggested by a previous report (Hamad *et al*, 2002), and, certainly, the clinical behaviour of human bladder tumours responding to genistein treatment is likely to more closely resemble that of the T24 cell line which was derived from human bladder TCC.

In summary, this study provided data of cell proliferation experiments employing antisense ODNs and protein kinase inhibitors, which indicate that the resistance of T24 cells to the genistein growth-inhibitory effect is predominantly due to the activation of oncogenic H-ras that transduces the growth signal through the MEK/ERK pathway and interferes with the JNK signalling pathway.
